# Reliable Diagnostics of SARS-CoV-2 Infections Using One- and Two-Gene Molecular Tests for a Viral RNA Detection—Results Questioning Previous Observations

**DOI:** 10.3390/diagnostics11101839

**Published:** 2021-10-05

**Authors:** Tomasz Bogiel, Mateusz Rzepka, Dagmara Depka

**Affiliations:** Microbiology Department, Ludwik Rydygier Collegium Medicum in Bydgoszcz, Nicolaus Copernicus University in Toruń, 9 Maria Skłodowska-Curie Street, 85-094 Bydgoszcz, Poland; mateusz.rzepka@cm.umk.pl (M.R.); dagmaradepka@cm.umk.pl (D.D.)

**Keywords:** 2019-nCoV, BD MAX™ System, COVID-19, *E* gene, *ORF1ab* gene, RNA detection, SARS-CoV-2, *S* gene

## Abstract

SARS-CoV-2 is a new virus from the *Coronaviridae* family and its rapid spread is now the most important medical problem worldwide. Currently used tests vary in the number and selection of SARS-CoV-2 target genes. Meanwhile, the choice of the appropriate target gene may be important in terms of a reliable detection of a viral RNA. As some researchers questioned the sensitivity of the monogenic VIASURE SARS-CoV-2 *S* gene Real Time PCR Detection Kit (CerTest Biotec, Zaragoza, Spain) in mid-2020, the aim of the study was to evaluate the usefulness of this kit, used along with the BD MAX™ System (Becton Dickinson, East Rutherford, NJ, USA), and compare the results with two-gene Bosphore Novel Coronavirus (2019-nCoV) Detection Kit v1 (Anatolia Diagnostics and Biotechnology Products Inc., Istanbul, Turkey). Both tests were carried out on 306 nasopharyngeal/oropharyngeal swabs. The consistent results (72 positive and 225 negative results found simultaneously in both kits) were obtained for 297 (97.1%) samples altogether, while discrepancies between the results of the evaluated tests were observed for nine (2.9%) specimens. There were no statistically significant differences between the method used and the frequency of positive results. Both tests, targeted at detecting one and two genes, are effective in SARS-CoV-2 RNA detection.

## 1. Introduction

The COVID-19 (Coronavirus disease 2019) pandemic announced on March 11, 2020 by the World Health Organization (WHO) has caused significant changes in almost every area of life [1,2,3]. Many medical laboratories worldwide have been adapted to run molecular diagnostics of a new virus from the *Coronaviridae* family—SARS-CoV-2 (Severe Acute Respiratory Syndrome Coronavirus 2), and various institutions have recognized viral RNA detection as the gold standard of COVID-19 diagnosis [4,5,6].

SARS-CoV-2 virus is transmitted primarily through close direct contact with an infected person and through the exchange of droplets and nanodroplets (e.g., coughing or sneezing by an infected person) [3]. The formation of aerosols (airborne) during medical procedures is also possible. SARS-CoV-2 genetic material has also been found on the surfaces of objects, in blood, urine and stool samples. However, it has not yet been proven that the spread occurs also via these routes [7,8,9,10].

SARS-CoV-2 infection manifests itself mainly in the respiratory system, but many people may not present any symptoms (asymptomatic carriers) [7,11,12].

The growing number of infections and mortality requires a rapid reduction of SARS-CoV-2 transmission. To achieve this goal, reliable laboratory diagnosis of infections involving this virus is essential. Currently, many different molecular tests are used for this purpose, including those adapted to automated systems. Reagents used in real-time RT-PCR (reverse-transcription polymerase chain reaction) use primers targeting different regions of the SARS-CoV-2 genome. The most commonly targeted viral genes include the *ORF1ab*, *N*, *S* and *E* genes [3,13,14,15,16,17,18]. According to the WHO recommendations, in order to confirm a case of SARS-CoV-2 infection in a laboratory, at least one gene should be detected in the areas with an established COVID-19 circulation or at least two genes in the areas where the virus circulation is unknown [4].

In the middle of 2020, some researchers commented on the lack of sensitivity of VIASURE SARS-CoV-2 *S* gene Real Time PCR Detection Kit (CerTest Biotec, Zaragoza, Spain) [19]. It was a very alarming announcement, since BD MAX™ Systems (Becton Dickinson, East Rutherford, NJ, USA) were used in a number of microbiological laboratories worldwide and the CerTest Biotec reagents were (and still are, to our knowledge) the only ones accompanying this device with regard to SARS-CoV-2 detection. As one of the first laboratories in Europe (and definitely the first in Poland) to use this system for SARS-CoV-2 detection, we were very concerned about this fact.

It is well known that the spread of SARS-CoV-2 largely depends on patients for whom the false-negative microbiological test results were obtained [20,21]. Thus, in this study we focused on the aforementioned evaluation approach in terms of the test sensitivity, specificity, and comparison of two tests for the diagnosis of SARS-CoV-2 infections: the indicated monogenic VIASURE SARS-CoV-2 *S* gene Real Time PCR Detection Kit used along with BD MAX™ System, and the two-gene Bosphore Novel Coronavirus (2019-nCoV) Detection Kit v1 (Anatolia Diagnostics and Biotechnology Products Inc., Istanbul, Turkey). The advantages and disadvantages of single and double gene tests were also highlighted.

## 2. Materials and Methods

### 2.1. Clinical Samples

The tests were carried out on 306 nasopharyngeal/oropharyngeal swabs collected between September 2020 and January 2021. They were preserved in dedicated vials or universal transport media (VTMs/UTMs) of three companies (UTM Universal Transport Medium, Copan, Brescia, Italy; Virus Transport and Preservation Medium, Biocomma^®^, Shenzhen, China; Virus Sample Stabilizer, Vazyme, Nanjing, China), each in a volume of 3 mL. The specimens were isolated from different patients or health care workers (one sample per patient). All the samples have been submitted for a routine diagnostics purpose to the clinical microbiology laboratory of Dr. Antoni Jurasz of the University Hospital No. 1 in Bydgoszcz, Poland.

### 2.2. VIASURE SARS-CoV-2 S Gene Test

The first part of the study involved a closed molecular system, which combines RNA extraction (using BD MAX™ ExK TNA-3 kit, Becton Dickinson, East Rutherford, NJ, USA) and detection. Both steps were performed in the BD MAX™ platform. 200 microliters of clinical material were used for the integrated nucleic acids extraction system that uses the defined extract volume for further detection within the same platform. Real-time RT-PCR reactions were performed using VIASURE SARS-CoV-2 *S* gene Real Time PCR Detection Kit; the *S* gene was a targeted in RT-PCR applying the device programmed protocol. Briefly, the amplification profile was: 15 min at 45 °C, 2 min at 98 °C and 45 cycles, each consisting of 10 s at 95 °C and 58 s at 60 °C. All the mentioned procedures were done according to the manufacturer’s instructions. The detailed information for all the applied kits is set in Supplementary material (Table S1).

### 2.3. RNA Extraction

The second part of the study was performed successively—the clinical samples stored at 4 °C for no longer than overnight, according to VTM/UTM manufacturer’s recommendations, and were used afterwards for RNA isolation. 140 microliters of material was used for the purposes of extraction using QIAamp Viral RNA Mini Kit (QIAGEN, Hilden, Germany) and QIAcube Connect device (QIAGEN, Hilden, Germany).

### 2.4. Bosphore Novel Coronavirus (2019-nCoV) Detection Kit v1

Bosphore Novel Coronavirus (2019-nCoV) Detection Kit v1 and the cobas z480 analyzer (Roche, Basel, Switzerland) were used to perform the real-time RT-PCR reaction. A volume of 10 microliters of RNA was applied for the *ORF1ab* and *E* genes detection, each targeted in one of the two separate RT-PCR reactions, performed at the same time with the addition of two separate reaction master mixes. The amplification profile was: 17 min at 50 °C, 6 min at 95 °C and 38 cycles, each consisting of 15 s at 97 °C and 1 min 10 s at 55 °C. The amplification product detected at the wavelength of 465–510 nm in both of the reactions, performed separately, was interpreted as positive results for both (*ORF1ab* and *E*) genes. The amplification curves detected at the wavelength of 533–580 nm corresponded to the internal control signal that was positive for all of the samples tested, in both of the reactions. The examples of the amplification curves obtained for the SARS-CoV-2 detection using Bosphore Novel Coronavirus kit (2019-nCoV) Detection Kit v1 are set in Supplementary material (Figure S1).

### 2.5. Data Interpretation, Results Confirmation and Verification

All the reactions with the divergent results between these methods were repeated to confirm the results repeatability. Additionally, all the inconsistent results obtained between the evaluated tests were verified with the application of Vitassay qPCR SARS-CoV-2 (Vitassay Healthcare S.L.U., Huesca, Spain) on the following day. The RNA, meanwhile, was kept at −20 °C and used in the volume of 5 microliters per each reaction. The verification test presented the highest sensitivity and was performed also on the cobas z480 analyzer using the RNA extracted with the application of QIAamp Viral RNA Mini Kit and QIAcube Connect device. The amplification profile was: 15 min at 45 °C, 2 min at 95 °C and 45 cycles, each consisting of 10 s at 95 °C and 50 s at 60 °C. The amplification product detected at the wavelength of 465–510 nm was interpreted as positive results for *ORF1ab*, 533–580 nm—*N* gene. The internal control amplification was confirmed at the wavelength of 533–610 nm for every sample. Each run was followed by color compensation of the obtained fluorescence results, done according to manufacturer’s instructions. The examples of the amplification curves obtained for the SARS-CoV-2 detection using Vitassay qPCR SARS-CoV-2 in the Supplementary materials (Figure S2).

For the classification of the samples into positive or negative, the analysis of the obtained amplification curves were done for each sample, also with respect to a particular gene in the Bosphore Novel Coronavirus (2019-nCoV) Detection Kit v1 and Vitassay qPCR SARS-CoV-2.

The declared sensitivities of the applied tests were as follows: Vitassay qPCR SARS-CoV-2 kit (≥10 virus copies per reaction), VIASURE SARS-CoV-2 *S* gene Real Time PCR Detection Kit (≥24 copies per reaction), and Bosphore Novel Coronavirus (2019-nCoV) Detection Kit v1 (≥18 copies per reaction). The corresponding C_T_ cut off values for a classification of the samples as positive in a particular test were as follows: 38, 40 and 33.

All the kits used in this study (for RNA isolation and SARS-CoV-2 RNA detection purpose) were certified for in vitro diagnostics (IVD certificate) by the manufacturers.

### 2.6. Quality Control

The study also involved laboratory validation of the tested kits, including the use of an outside, commercially available laboratory quality controls (Labquality, Helsinki, Finland). Both tests applied in the present study were verified using three samples with unknown SARS-CoV-2 status, obtained during External Quality Assessment Lab Quality Programme 2021 (“blinded” samples, labelled as S001-3) called “SARS-CoV-2, nucleic acid detection” and the compatibility of the results was confirmed. The aforementioned samples, serving as quality control material, were used according to the stated programme instructions. They were treated in the same way as the clinical specimen.

### 2.7. Statistical Analysis

The sensitivity and specificity of the tests were calculated using PQStat for Windows, version 1.8.2 (PQStat Software, Poznan, Poland). The inconclusive results were classified as true positive if a positive result for a given test sample was obtained also with the application of Vitassay qPCR SARS-CoV-2 kit.

Statistical differences between the assays in the frequency of positive results were calculated with the application of χ^2^ test; *p* < 0.05 was considered as statistically significant.

## 3. Results 

The classification of the sample as positive or negative for SARS-CoV-2 RNA was performed based on the analysis of the obtained values of the cycle threshold (C_T_) and amplification curves shapes. Regardless of the assay used, 228 (74.5%) samples were negative (called true negative). A similar number of positive results (78 and 73) was obtained in the individual method (Table 1). There were no statistically significant differences (χ² = 0.1236) between the assays used and the frequency of positive results (*p* = 0.7252). 

However, only the results that were positive in both methods were considered as true positive. Thus, five (1.6%) inconclusive results were found using the Bosphore Novel Coronavirus (2019-nCoV) Detection Kit v1, which means that the presence of only one of the two detected genes was confirmed (Table 1).

For 9 (2.9%) samples, the results were divergent between the assays, as shown in Table 2.

For all samples with different results, the RT-PCR reactions were repeated with each detection kit. Additionally, these samples were tested with the Vitassay qPCR SARS-CoV-2 kit. The results that were consistent in at least two methods applied were considered to be true. The distribution of five different genes detected using these tests is presented in Table 3.

Based on the data shown and the chosen criteria, the results revealed three false-negative samples (cross contamination, perhaps) and two false-positive samples obtained using the VIASURE SARS-CoV-2 *S* gene Real Time PCR Detection Kit, while one false negative and five inconclusive results occurred with the Bosphore Novel Coronavirus kit (2019-nCoV) Detection Kit v1.

**Figure 1:**
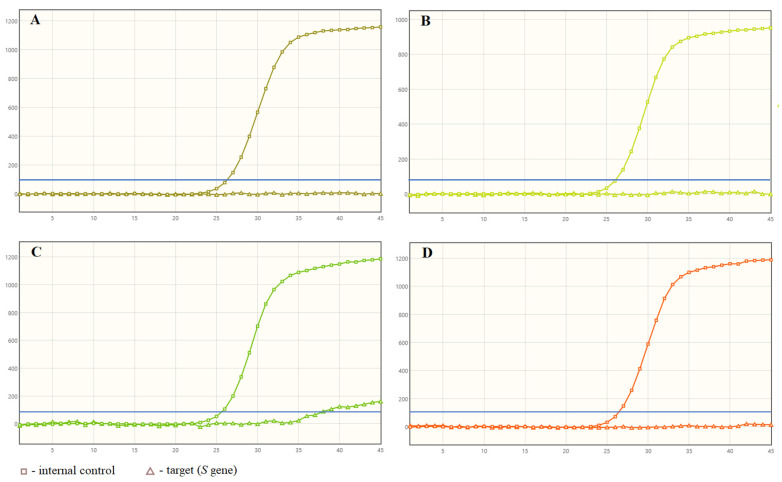
The examples of false negative results from BD MAX™ System (**A**–**D**); blue lines represent the threshold line.

Figure 1A,B shows the false negative results obtained with BD MAX™ System. One of the samples (Figure 1C) showed a non-specific shape of the amplification curve and a slight increase in fluorescence above the threshold line. The result of the second reaction for this samples was interpreted as negative (Figure 1D), while *ORF1ab*, *E* and *N* genes were detected using both the remaining kits tested.

**Figure 2:**
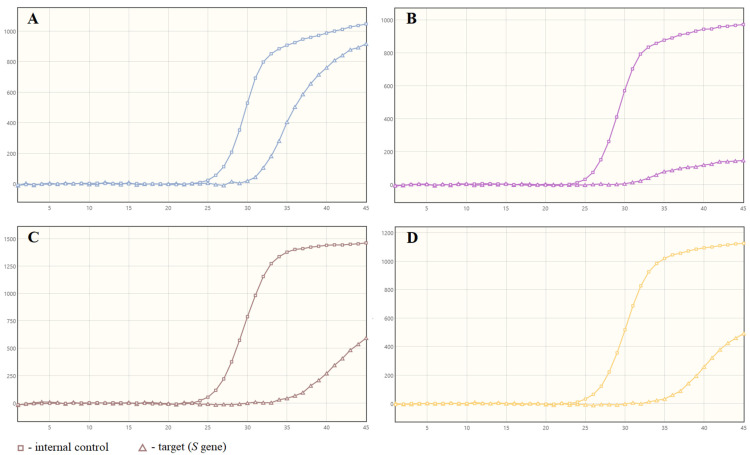
True positive results on BD MAX™ System which were inconclusive (**A**–**C**) or negative (**D**) using Bosphore Novel Coronavirus kit (2019-nCoV) Detection Kit v1.

Four specimens were positive on the BD MAX™ System, while with Bosphore Novel Coronavirus (2019-nCoV) Detection Kit v1, three were inconclusive and one was negative. The increase of fluorescence for these samples, with C_T_ values ranging from 34.7 to 37.3, was significantly lower than for most of the positive samples (Figure 2).

## 4. Discussion and Conclusions

For over a year of the SARS-CoV-2 pandemic, there was a constant need for molecular tests to detect the genetic material of the virus. This was due to its global spread and emergence of new SARS-CoV-2 variants [22,23,24]. Serological diagnostics are currently limited due to the inability to detect the infection at its early stages. They are primarily used for epidemiological purposes and for the testing of the immune system response after infection and/or vaccination [25,26]. This underlines the necessity of using reliable tests that are based on RT-PCR.

In the available literature there are a number of studies which describe or compare the diagnostic approaches and parameters of SARS-CoV-2 RNA detection kits [10,27,28,29,30,31]. However, there are only a few concerning the diagnostic usefulness of the VIASURE assay [19,32] with only one referring to the version of the test evaluated in our study [19]. Noteworthy in the mentioned study is that the sensitivity of the VIASURE test, which is targeted for the *S* gene, was questionable.

In the present study we showed that, regardless of the test used (single or double-gene test), it was not possible to correctly determine the viral status of all samples. However, a high agreement (97.1%) was found in the obtained results, obtaining only nine (2.9%) discrepant results. Despite the lack of statistically significant differences between the method used and the frequency of positive results, the identified discrepancies might have significant epidemiological consequences, especially with regard to false negative results. Thus, a reliable test result is crucial in the diagnosis of patients and allows for the reduction of virus transmission, which is also of economic importance.

The use of the BD MAX™ System, which combines RNA extraction and real-time RT PCR, provides results in a shorter time. Depending on the number of samples tested, the time does not exceed three hours, including the sample preparation stage. In most cases, a specific shape of the amplification curves is obtained for the positive results. Research by Navarathna et al. [21] showed that this system interprets many samples as positive, even though they are actually negative. This is mostly due to the non-specific shape of the amplification curves which cross the threshold line. In our study, only two such plots were obtained (Figure 1C and Figure 2B). Both of these samples were SARS-CoV-2 true positive; however, one of them was a false negative when repeating the reaction on the BD MAX™ System afterwards (Figure 1D). It might have resulted from the low number of RNA copies in the specimen or from RNA stability in the sample. This situation underlines the necessity of careful interpretation of the obtained results and verification of the status of such samples using other diagnostic kits. We also found a high sensitivity in the VIASURE SARS-CoV-2 *S* gene Real Time PCR Detection Kit and thus a very low number of false-negative results, which is opposite to the observations reported by other researchers [19].

However, using the BD MAX™ System, two false-positive results were obtained in the first test, which turned out to be negative after repeated testing. In both of these situations, during the first testing, the same reaction showed the presence of other highly positive SARS-CoV-2 RNA samples. Despite the great care and experience in molecular biology research, samples might have been contaminated during their initial preparation. Contamination related to the operation at the initial point of investigation within the device cannot be ruled out, although it seems unlikely.

Only one false negative and five (1.6%) ambiguous results were obtained with the Bosphore Novel Coronavirus (2019-nCoV) Detection Kit v1. In addition, we observed that the *E* gene was not detected in the samples with equivocal results while the *ORF1ab* gene was still present. These observations are consistent with the results of studies by other authors [33,34]. A possible explanation of this situation is the occurrence of a mutation in the nucleotide sequence of this gene for the used fluorescent primers or probes [34,35,36]. In such a situation, we suggest repeating the reaction using tests targeting other regions of the SARS-CoV-2 genome. It should be noted that the *S* gene was not detected in the two equivocal samples, which would result in the classification of these samples as negative when using the monogenic test only.

Quality control samples applied in this study were treated in the same manner as the investigated samples, and the consistency of the results for all samples was confirmed with all the methods used in this study. This fact additionally underlines the reliability of the results obtained in the present study. Moreover, a previous study indicates that VIASURE SARS-CoV-2 Real Time PCR Detection Kits is a reliable method for SARS-CoV-2 RNA detection [32]. However, the mentioned research was performed on a newer variant of the assay, targeting a different gene (*N1* and *N2*).

Our study confirms that the use of two-gene tests increases the sensitivity and specificity of the test (98.7% and 100%, respectively) compared to the monogenic one (96.2% and 99.1%, respectively). Reduction of the possibility of obtaining false-negative results with the use of high diagnostic sensitivity tests is particularly important in limiting SARS-CoV-2 outbreaks. There is no doubt that the differences in the detection limits of each kit and target genes have an impact on the obtained values of diagnostic sensitivity and the specificity of the investigated tests.

The limitations of the study are that the amplification protocols are not the same for each test; different volumes of clinical samples were applied for RNA extraction (200 microliters for BD MAX™ System vs. 140 microliters for QIAcube Connect device) and different protocols of genetic material isolation were used for a particular assay. Thus, this might have influenced the sensitivity of the results, especially for the samples with relatively low viral load. It is worth noting that the BD MAX™ System is a closed molecular IVD platform and it was not possible to modify the device settings to unify the sample volume or other extraction conditions. It was also impossible to use the RNA derived from it in the following steps for the purpose of further research.

The tests detecting one gene are mostly cheaper than those detecting at least two SARS-CoV-2 genes. However, the use of the latter can reduce the rate of false-negative results [20,21]. This diagnostic approach increases the probability of viral genetic material detection, including, for example, the sequences with genes mutations [37,38]. Currently, this is particularly important due to the observed dynamically increasing number of new SARS-CoV-2 variants [22,23]. The unquestionable advantage of monogenic tests is that the interpretation criteria of the obtained results are very certain. The use of multigene tests may generate equivocal results, which require repeating of the test with the use of other molecular kits, thus extending the time required for the diagnostic procedure. Regardless of the type of test used in a given laboratory, one should always remember the importance of reliable results in a patient’s diagnosis.

## Figures and Tables

**Table 1:** Comparison of the results obtained with VIASURE SARS-CoV-2 *S* gene Real Time PCR Detection Kit and Bosphore Novel Coronavirus (2019-nCoV) Detection Kit v1 (*n* = 306).

	Results			Sensitivity (%)	Specificity (%)
	(+)	(−)	IR		
VIASURE SARS-CoV-2 *S* gene Real Time PCR Detection Kit	78 (25.5%)	228 (74.5%)	NA	96.2	99.1
Bosphore Novel Coronavirus (2019-nCoV) Detection Kit v1	73 (23.9%)	228 (74.5%)	5 (1.6%)	98.7	100

(+)—positive result, (−)—negative result, IR—inconclusive result, NA—not applicable.

**Table 2:** Compatibility of results between the applied methods (*n* = 306).

		VIASURE SARS-CoV-2 *S* Gene Real Time PCR Detection Kit		
		(+)	(−)	IR
*Bosphore Novel Coronavirus (2019-nCoV) Detection Kit v1*	(+)	72	1	NA
	(−)	3 ^a^	225	NA
	IR	3 ^b^	2	NA

(+)—positive result, (−)—negative result, IR—inconclusive result; ^a^—two of the samples were positive in the first reaction but negative in the repeat with VIASURE SARS-CoV-2 *S* gene Real Time PCR Detection Kit, ^b^—one of the samples were negative in the first reaction but positive in the repeat with VIASURE SARS-CoV-2 *S* gene Real Time PCR Detection Kit, NA—not applicable.

**Table 3:** Distribution of the divergent results for the gene sequences detected in the individual methods (*n* = 9).

*n*	%	VIASURE	Bosphore		Vitassay	
		*S* Gene	*ORF1ab* Gene	*E* Gene	*ORF1ab* Gene	*N* Gene
3	1	+	+	−	+	+
2 ^a^	0.6	+	−	−	−	−
2	0.6	−	+	−	+	+
1	0.3	+	−	−	+	+
1	0.3	−	+	+	+	+

(+)—positive result, (−)—negative result, *n*—number of samples, ^a^—two samples were positive in the first reaction but negative in the repeat with VIASURE SARS-CoV-2 S gene Real Time PCR Detection Kit.

## Data Availability

Data presented in this study are available upon request. “External quality assessment service agreement terms and conditions” do not prohibit information about the participation of a specific medical laboratory in the Quality Control Program. Information on the participation of individual medical laboratories in quality control programs and certificates in this regard are available to the public as full documentation of the laboratory and can be made available on request.

## Supplementary Materials

The following are available online at https://www.mdpi.com/article/10.3390/diagnostics11101839/s1, Table S1. Minimum Information for Publication of Quantitative Real-Time PCR Experiments; Figure S1. Amplification curves obtained for the SARS-CoV-2 detection using Bosphore Novel Coronavirus kit (2019-nCoV) Detection Kit v1; Figure S2. Amplification curves obtained for the SARS-CoV-2 detection using Vitassay qPCR SARS-CoV-2.
