# Supplementary material for: Reliable Diagnostics of SARS-CoV-2 Infections Using One- and Two-Gene Molecular Tests for a Viral RNA Detection—Results Questioning Previous Observations
Source: Diagnostics (Basel). 2021 Oct 5;11(10):1839. doi: 10.3390/diagnostics11101839 (PMC8534906; doi:10.3390/diagnostics11101839)

Table S1. Minimum Information for Publication of Quantitative Real-Time PCR Experiments

**EXPERIMENTAL DESIGN**	
**Definition of experimental and control groups**	The tests were carried out on 306 nasopharyngeal/oropharyngeal swabs collected between September 2020 and January 2021 into dedicated viral transport media (VTM) of three companies (UTM Universal Transport Medium, Copan, Virus Transport and Preservation Medium, Biocomma® and Virus Sample Stabilizer, Vazyme), each in a volume of 3 ml. The specimens were isolated from different patients or health care workers (one sample per patient). All samples have been submitted for routine diagnostics.
**Number within each group**	306 nasopharyngeal/oropharyngeal swabs.
**Assay carried out by core lab or investigator's lab?**	Investigator's lab.
**Acknowledgement of authors' contributions**	not applicable
**SAMPLE**	
**Description**	
Volume/mass of sample processed	BD MAX™ ExK™ TNA-3 (Becton Dickinson): 200 microliters<br>QIAamp Viral RNA Mini Kit (QIAGEN) and QIAcube Connect device (QIAGEN): 140 microliters.
Microdissection or macrodissection	not applicable
**Processing procedure**	
If frozen - how and how quickly?	not applicable
If fixed - with what, how quickly?	not applicable
**Sample storage conditions and duration (especially for FFPE samples)**	The clinical samples stored at 4°C for 1 to 3 hours, according to UTM manufacturer’s recommendations were used afterwards for RNA isolation with BD MAX™ ExK™ TNA-3 (Becton Dickinson). The clinical samples stored no longer than overnight at 4°C, according to VTM manufacturer’s recommendations were used afterwards for RNA isolation with QIAamp Viral RNA Mini Kit (QIAGEN) and QIAcube Connect device (QIAGEN).
**NUCLEIC ACID EXTRACTION**	
**Procedure and/or instrumentation**	
Name of kit and details of any modifications	BD MAX™ ExK™ TNA-3 (Becton Dickinson) and QIAamp Viral RNA Mini Kit (QIAGEN).
Source of additional reagents used	not applicable
**Details of DNase or RNAse treatment**	not applicable
**Contamination assessment (DNA or RNA)**	Negative controls in each run, incompatible reactions between methods repeated.
**Nucleic acid quantification**	
Instrument and method	BD MAX™ system (Becton Dickinson) and QIAcube Connect device (QIAGEN).
Purity (A260/A280)	not applicable
Yield	not applicable
**RNA integrity method/instrument**	
RIN/RQI or Cq of 3' and 5' transcripts	not applicable
Electrophoresis traces	not applicable
**Inhibition testing (Cq dilutions, spike or other)**	An inhibition of the PCR reaction excluded by the amplification of internal control.

	In case of absence of internal control signal in sample it is recommended to repeat the assay diluting the sample 1:10 to check for possible problems of inhibition.
**REVERSE TRANSCRIPTION**	
**Complete reaction conditions**	
Amount of RNA and reaction volume	VIASURE SARS-CoV-2 *S gene* Real Time PCR Detection Kit (CerTest Biotec): data not provided by the manufacturer (closed molecular platform).<br>Bosphore Novel Coronavirus (2019-nCoV) Detection Kit v1 (Anatolia Geneworks): 10 microliters.<br>Vitassay qPCR SARS-CoV-2 (Vitassay Healthcare S.L.U.): 5 microliters.
Priming oligonucleotide (if using GSP) and concentration	data not provided by the manufacturer
Reverse transcriptase and concentration	data not provided by the manufacturer
Temperature and time	VIASURE SARS-CoV-2 *S gene* Real Time PCR Detection Kit (CerTest Biotec): 15 min at 45°C.<br>Bosphore Novel Coronavirus (2019-nCoV) Detection Kit v1 (Anatolia Geneworks): 17 min at 50°C.<br>Vitassay qPCR SARS-CoV-2 (Vitassay Healthcare S.L.U.): 15 min at 45°C.
Manufacturer of reagents and catalogue numbers	VIASURE SARS-CoV-2 *S gene* Real Time PCR Detection Kit (CerTest Biotec): BD REF 444212.<br>Bosphore Novel Coronavirus (2019-nCoV) Detection Kit v1 (Anatolia Geneworks): ABCOW3.<br>Vitassay qPCR SARS-CoV-2 (Vitassay Healthcare S.L.U.): 7081046.
**Cqs with and without RT**	not applicable
**Storage conditions of cDNA**	not applicable
**qPCR TARGET INFORMATION**	
**If multiplex, efficiency and LOD of each assay.**	VIASURE SARS-CoV-2 *S gene* Real Time PCR Detection Kit (CerTest Biotec): has a detection limit of ≥ 24 cDNA copies per reaction (cp/rxn) with a positive rate of ≥ 95%.<br>Bosphore Novel Coronavirus (2019-nCoV) Detection Kit v1 (Anatolia Geneworks): has a detection limit of ≥ 18 cDNA copies per reaction (cp/rxn).<br>Vitassay qPCR SARS-CoV-2 (Vitassay Healthcare S.L.U.): has a detection limit of ≥ 10 cDNA copies per reaction (cp/rxn).
**Sequence accession number**	data not provided by the manufacturer
**Location of amplicon**	
Amplicon length	data not provided by the manufacturer
In silico specificity screen (BLAST, etc)	data not provided by the manufacturer
Pseudogenes, retropseudogenes or other homologs?	not applicable
Sequence alignment	not applicable
Secondary structure analysis of amplicon	data not provided by the manufacturer
**Location of each primer by exon or intron (if applicable)**	not applicable
**What splice variants are targeted?**	data not provided by the manufacturer
**qPCR OLIGONUCLEOTIDES**	
**Primer sequences**	data not provided by the manufacturer

**RTPrimerDB Identification Number**	data not provided by the manufacturer
**Probe sequences**	data not provided by the manufacturer
**Location and identity of any modifications**	data not provided by the manufacturer
**Manufacturer of oligonucleotides**	data not provided by the manufacturer
**Purification method**	data not provided by the manufacturer
**qPCR PROTOCOL**	
**Complete reaction conditions**	
Reaction volume and amount of cDNA/DNA	VIASURE SARS-CoV-2 *S gene* Real Time PCR Detection Kit (CerTest Biotec): data not provided by the manufacturer (automatic method). Bosphore Novel Coronavirus (2019-nCoV) Detection Kit v1 (Anatolia Geneworks): reaction volume - 25 microliters. Vitassay qPCR SARS-CoV-2 (Vitassay Healthcare S.L.U.): reaction volume - 20 microliters.
Primer, (probe), Mg++ and dNTP concentrations	data not provided by the manufacturer
Polymerase identity and concentration	data not provided by the manufacturer
Buffer/kit identity and manufacturer	data not provided by the manufacturer
Exact chemical constitution of the buffer	data not provided by the manufacturer
Additives (SYBR Green I, DMSO, etc.)	data not provided by the manufacturer
**Manufacturer of plates/tubes and catalog number**	BD MAX™ PCR Cartridges (Becton Dickinson, Catalog No. 437519); LightCycler® 480 Multiwell Plate 96, white (Roche, Catalog No. 04729692001); SARS-CoV-2 strips (Catalog No. 7041S046/7042S046).
**Complete thermocycling parameters**	VIASURE SARS-CoV-2 *S gene* Real Time PCR Detection Kit (CerTest Biotec): 2 min at 98°C and 45 cycles, each consisting of 10 sec at 95°C and 58 sec at 60 °C. Bosphore Novel Coronavirus (2019-nCoV) Detection Kit v1 (Anatolia Geneworks): 6 min at 95°C and 38 cycles, each consisting of 15 sec at 97°C and 1 min 10 sec at 55°C. Vitassay qPCR SARS-CoV-2 (Vitassay Healthcare S.L.U.): 2 min at 95°C and 45 cycles, each consisting of 10 sec at 95°C and 50 sec at 60°C.
**Reaction setup (manual/robotic)**	VIASURE SARS-CoV-2 *S gene* Real Time PCR Detection Kit – integrated robotic reaction setup; Bosphore Novel Coronavirus (2019-nCoV) Detection Kit v1 (Anatolia Geneworks) and Vitassay qPCR SARS-CoV-2 (Vitassay Healthcare S.L.U.) – manual reaction setup.
**Manufacturer of qPCR instrument**	BD MAX™ System (Becton Dickinson); cobas z480 Instrument II (Roche).
**qPCR VALIDATION**	
**Evidence of optimisation (from gradients)**	data not provided by the manufacturer
**Specificity (gel, sequence, melt, or digest)**	data not provided by the manufacturer
**For SYBR Green I, Cq of the NTC**	data not provided by the manufacturer
**Standard curves with slope and y-intercept**	
PCR efficiency calculated from slope	not applicable
Confidence interval for PCR efficiency or standard error	not applicable

r2 of standard curve	not applicable
**Linear dynamic range**	
Cq variation at lower limit	not applicable
Confidence intervals throughout range	not applicable
**Evidence for limit of detection**	data not provided by the manufacturer
**If multiplex, efficiency and LOD of each assay.**	VIASURE SARS-CoV-2 S gene Real Time PCR Detection Kit (CerTest Biotec): has a detection limit of ≥ 24 cDNA copies per reaction (cp/rxn) with a positive rate of ≥ 95%.<br>Bosphore Novel Coronavirus (2019-nCoV) Detection Kit v1 (Anatolia Geneworks): has a detection limit of ≥ 18 cDNA copies per reaction (cp/rxn).<br>Vitassay qPCR SARS-CoV-2 (Vitassay Healthcare S.L.U.): has a detection limit of ≥ 10 cDNA copies per reaction (cp/rxn).
**DATA ANALYSIS**	
**qPCR analysis program (source, version)**	BD MAX™ System Software version: V5.14 A; LightCycler® 480 Software release 1.5.1.62 SP3, Version 1.5.1.62.
Cq method determination	not applicable
Outlier identification and disposition	not applicable
**Results of NTCs**	Negative, set in each run of Bosphore Novel Coronavirus (2019-nCoV) Detection Kit v1 (Anatolia Geneworks) and Vitassay qPCR SARS-CoV-2 (Vitassay Healthcare S.L.U.).
**Justification of number and choice of reference genes**	not applicable
**Description of normalisation method**	not applicable
**Number and concordance of biological replicates**	not applicable
**Number and stage (RT or qPCR) of technical replicates**	not applicable
**Repeatability (intra-assay variation)**	not applicable
**Reproducibility (inter-assay variation, %CV)**	not applicable
**Power analysis**	not applicable
**Statistical methods for result significance**	ABS Quant/Fit Points
**Software (source, version)**	LightCycler® 480 Software release 1.5.1.62 SP3, Version 1.5.1.62
**Cq or raw data submission using RDML**	not applicable

Figure S1. Amplification curves obtained for the SARS-CoV-2 detection using Bosphore Novel Coronavirus kit (2019-nCoV) Detection Kit v1; positive results for six clinical samples (green lines) and positive controls (blue lines) are observed at the corresponding wavelength (A - amplification curves for the *ORF1ab* gene; B - amplification curves for the internal control in the wells detecting the *ORF1ab* gene; C - amplification curves for the *E* gene; D - amplification curves for the internal control in the wells detecting the *E* gene); red curves represent negative controls of the test.

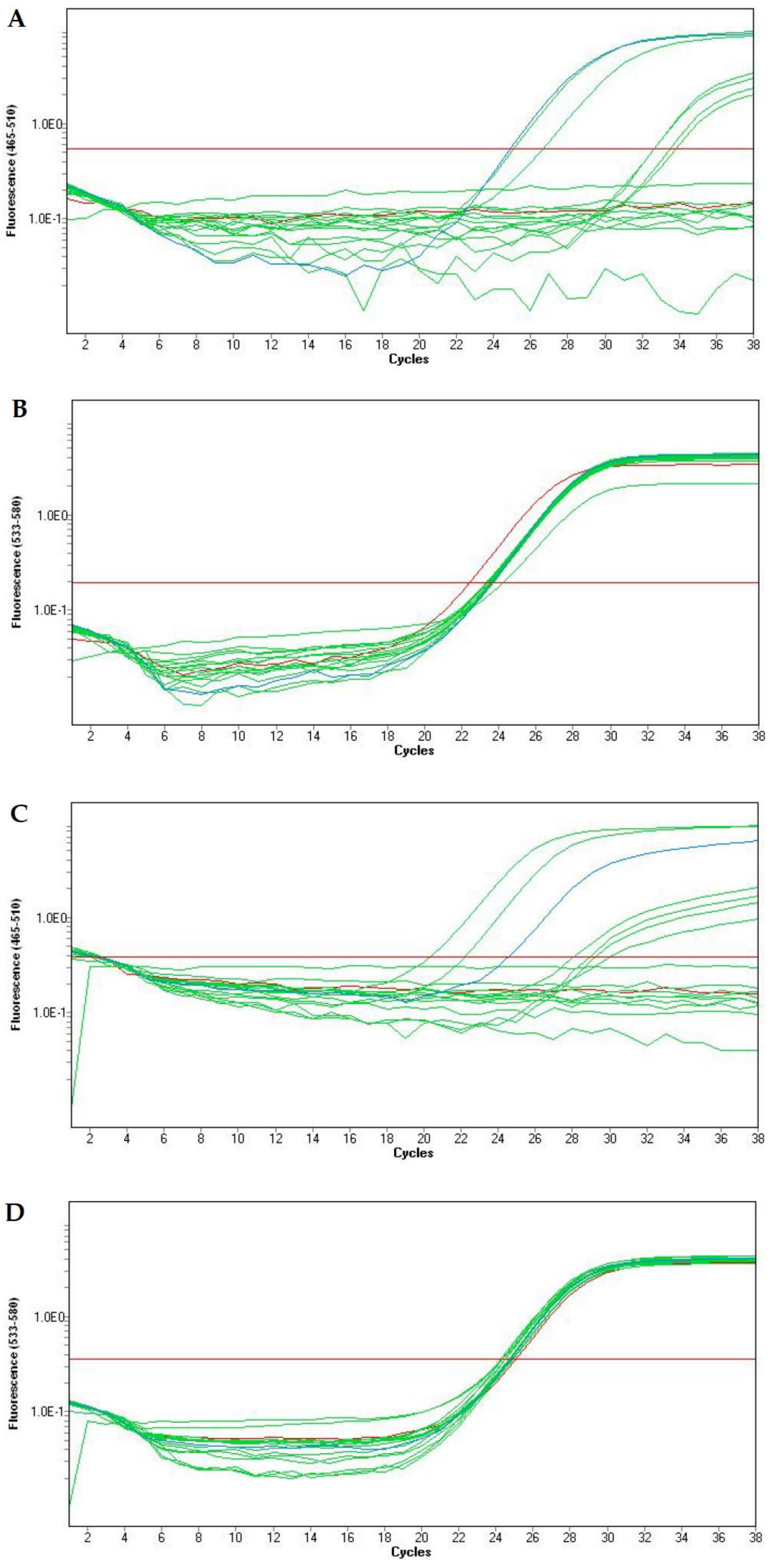

Figure S2. Amplification curves obtained for the SARS-CoV-2 detection using Vitassay qPCR SARS-CoV-2; positive results for one clinical sample (green lines) and positive controls (blue lines) are observed at the corresponding wavelength (A - amplification curves for the *ORF1ab* gene; B - amplification curves for the internal control in the wells detecting both *ORF1ab* and *N* genes; C - amplification curves for the *N* gene); red curves represent negative controls of the test.

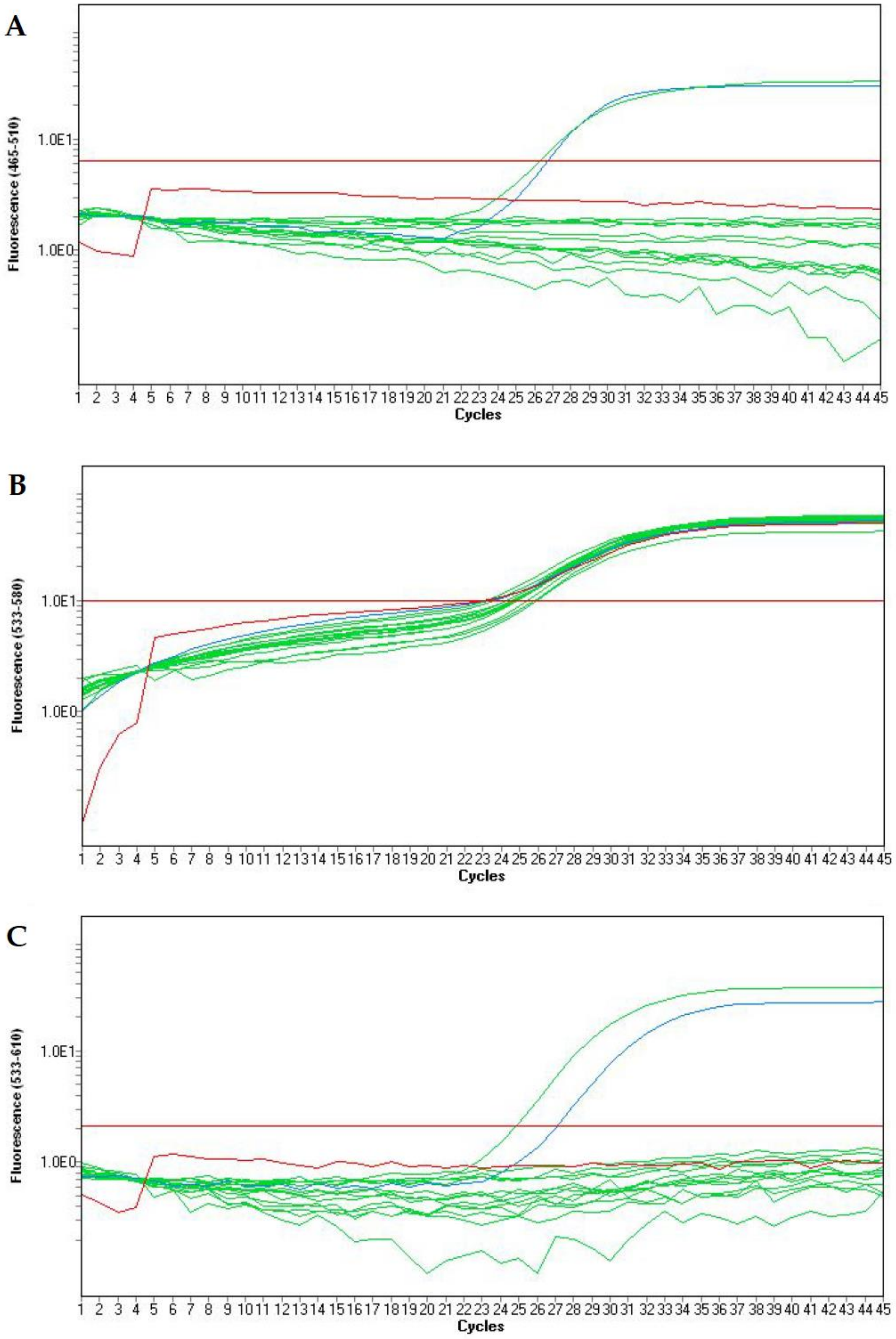

Supplement: Supplementary file 1 [file diagnostics-11-01839-s001.zip › diagnostics-1369036-supplementary.pdf]
